# Comparative evaluation of the effects of different methods of post space preparation in primary anterior teeth on the fracture resistance of tooth restorations

**DOI:** 10.15171/joddd.2019.022

**Published:** 2019-08-14

**Authors:** Bahman Seraj, Sara Ghadimi, Ebrahim Najafpoor, Fatemeh Abdolalian, razieh khanmohammadi

**Affiliations:** ^1^Dental Research Center and Department of Pediatric Dentistry, School of Dentistry, Tehran University of Medical Sciences, Tehran, Iran; ^2^Laser Research Center of Dentistry, Dental Research Institute, Department of Pediatric Dentistry, School of Dentistry, Tehran University of Medical Sciences, Tehran, Iran; ^3^Department of Pediatric Dentistry, School of Dentistry, Tabriz University of Medical Sciences, Tabriz, Iran; ^4^Department of Periodontics, School of Dentistry, Ahvaz Jundishapur University of Medical Sciences, Ahvaz, Iran; ^5^Department of Pediatric Dentistry, School of Dentistry, Tehran University of Medical Sciences, Tehran, Iran

**Keywords:** Chlorhexidine, fracture resistance, postoperative modalities, sodium hypochlorite

## Abstract

***Background.*** Severely damaged teeth do not have adequate structure to support the composite crown; therefore, use of the canal space has been suggested to increase retention. Furthermore, the effect of post space irrigation protocols on the fracture resistance of the primary anterior teeth has not definitely been studied in postoperative modalities. This study compared the fracture resistance of restorations of primary anterior teeth following irrigation of the post space with sodium hypochlorite and chlorhexidine with and without application of burs.

***Methods.*** Ninety-four extracted primary anterior teeth were selected. Root canal treatments were carried out, 4 mm of the post space was left empty and 1 mm was regarded as a bed. The post space was prepared with and without a bur and the irrigation procedures were carried out with 0.2% chlorhexidine and 2.5% sodium hypochlorite solutions. Composite posts were inserted into the canals and the crowns were restored. Following composite etching and light-curing, the fracture resistance of the restored teeth was determined by a universal testing machine.

***Results.*** In the sodium hypochlorite group, fracture resistance of the teeth was 376.8±107.29 N and 475.5±186.89 N without and with bur preparation, respectively. For chlorhexidine protocol, the values were 370.88±175.46 N and 430.85±178.22 N without and with bur preparation, respectively. The effect of irrigating material was not significant; however, the bur and irrigating preparation significantly increased the fracture resistance of the restored teeth (P=0.02).

***Conclusion.*** Post space irrigation with 0.2% chlorhexidine or 2.5% sodium hypochlorite did not significantly affect the fracture resistance of primary anterior teeth in the post treatment modality.

## Introduction

It is generally accepted that dental caries is the most common chronic childhood disease.^1,2^ Early childhood caries quickly involves the maxillary anterior teeth at an early age, and usually a large part of the tooth structure is lost when parents discover these caries and take the baby to a dentist. It has been a challenge to reconstruct severely damaged anterior teeth from several points of view, including difficulty in repairing and uncooperative children at an early age; therefore, tooth extraction has long been considered as a selective therapeutic approach for these cases, but many parents are dissatisfied and are asking for esthetic restorations.^3,4^

Some of the existing therapies are prefabricated crowns and composite restorations. In cases of severe tooth decay, it is necessary to carry out root canal therapy and place a post in the root canal prior to crown reconstruction.^4^ Since severely damaged teeth have insufficient coronal structure to support and retain composite crowns, it is recommended that the root canal space be used to enhance restoration retention.^5^

It is difficult to bond the root canal due to the characteristics and control problems of the adhesive systems, root canal anatomy, inaccessibility of entire area for complete root canal cleaning, tooth position, the presence of coronal residual tissue in different parts, and different techniques of light curing, as well as operator experience and skill. The canal walls after post space preparation are covered with a thick smear layer containing rough debris and gutta-percha or sealer residues, possibly interfering with effective bonding with dentin. Hence, it is important to prepare the post space in a manner to effectively remove the smear layer and the sealer to achieve proper access to dentin to create effective bonding with decalcified dentin. Simultaneously, various methods such as etching and use of chemical agents for irrigation, including NaOCl and EDTA or ultrasonic solutions have been suggested to remove the smear layer.

On the other hand, the application of sodium hypochlorite has been suggested due to its physicochemical and antibacterial properties and its tissue solubility in root canals.^6^ In addition, its use can result in changes in dentinal proteins and collagen fibers; these changes might interfere with the formation of a hybrid layer that plays an important role in resin‒dentin tensile bond strength. Some studies have concluded that sodium hypochlorite prevents the adhesion of resin through changes in dentin structure.^7^ Chlorhexidine has been proposed as an endodontic detergent due to its extensive antimicrobial effects, durability, biocompatibility and physiological properties. Chlorhexidine can prevent the degradation of collagen fibers and maintain the hybrid layer integrity.^8,9^

In this regard, most studies evaluated the effects of different preparation methods on the bond strength values of endodontic posts in permanent teeth. However, limited studies are available on deciduous teeth. Therefore, the present study aimed to compare the fracture resistance values of severely damaged primary anterior teeth using different methods of preparation of the coronal one-third of root canals to help clinicians select the most appropriate therapeutic method to repair these teeth using these findings.

## Methods

In this in vitro research, data were collected through laboratory tests. A total of 94 primary anterior teeth extracted because of severe caries and being non-restorable were collected and stored in 0.5% chloramine-T solution for a week and in distilled water in a refrigerator until completion of the specimen collection. The distilled water was renewed periodically.

The teeth were cut from 1 mm above the CEJ using a fissure bur (#245) in a high-speed turbine under water spray. The teeth were randomly divided into four groups by randomized block designs. To match the samples in the study groups, the diameters of the teeth in the CEJ region were measured by a caliper. The mean diameters in the four groups were not different significantly.

### Procedural Steps

The root canals were filed 1 mm shorter than the working length up to three files larger than the primary file (2.5% NaOCl was used to irrigate the canal while filing in the sodium hypochlorite groups and 0.2% chlorhexidine in the chlorhexidine groups). The root canals were dried with paper points and filled with ZOE (Kem Dent) 1 mm shorter than the working length.

The coronal 4-mm length of the root canal-filling material was removed to provide post space, and a 1-mm base of Light Dycal (Spident-base.it) was placed and light-cured for 40 seconds with an LED light-curing unit (Radi, SDI Co.). Excess materials were cleaned to provide a 3-mm post space.

The post space was prepared in each group as follows:

**Group 1:** The post space was prepared using a fissure bur in a high-speed handpiece so that the excess materials were removed. It was then irrigated with 0.2% chlorhexidine.

**Group 2:** The post space was irrigated with 0.2% chlorhexidine.

**Group 3:** The post space was prepared by a fissure bur in a high-speed handpiece so that the excess materials were removed. It was then irrigated with 2.5% sodium hypochlorite solution.

**Group 4:** The post space was irrigated with 2.5% hypochlorite solution.

**Figure 1:**
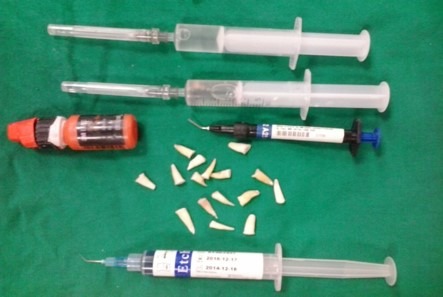
Bonding agent and flowable composite resin kit.

The following steps were performed on all the teeth: acid etching for 20 seconds and irrigation for 15 seconds, drying the tooth so that the dentin did not completely dry, application of two layers of bonding agent (Single Bond, 3M ESPE, USA) with a microbrush, drying gently for 2‒5 seconds and light-curing for 20 seconds, followed by incremental placement of flowable composite resin in two steps, light-curing in each step to prepare the post, and incremental formation of the crown at a height of 4 mm (each layer was <2 mm and finally light-cured for 40 seconds) (Figure 1).

All the samples were polished by composite resin polishing burs in a high-speed handpiece to create a uniform surface.

**Figure 2:**
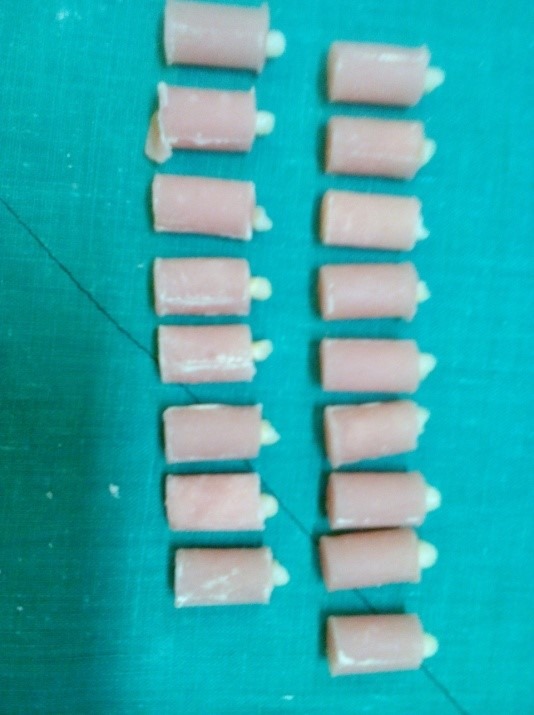
The specimens mounted in cylindrical acrylic resin

The specimens were then mounted in acrylic resin in a cylindrical mold 1 mm under the CEJ (Figure 2).

Subsequently, the samples were subjected to 1000 thermal cycles at 5/55°C.

To evaluate fracture resistance, the samples were placed in a universal testing machine (Zwick/Roell Z050 Germany) under a compressive force with an angle of 148° (35) and a crosshead speed of 1 mm/s.

**Figure 3:**
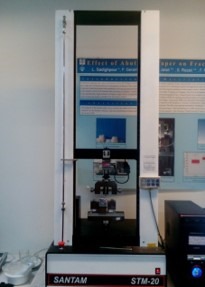
Universal testing machine.

The force applied in the middle third of the palatal surface continued until the restoration fractured (Figure 3). The specimens were examined after fracture to evaluate the fracture pattern by the operator and classified based on the fracture site as restorable (fractures above the CEJ) and non-restorable (fractures below the CEJ).

Data were analyzed with SPSS 21.0. Two-way ANOVA was used to analyze the effects of irrigation type and the use or non-use of a bur for post space preparation in primary anterior teeth. The type I error level was considered to be 0.05 (α=0.05) in the present study like other studies.

## Results

The fracture resistance values of the teeth were 376.08±107.29 N (with a range of 240.17‒670.32 N) in the presence of sodium hypochlorite solution (without a bur) and 475.05±186.89 N (with a range of 157.67±1020.93 N) in the presence of sodium hypochlorite solution (with a bur). On the other hand, fracture resistance values were 370.88±175.46 N (with a range of 112.58±664.97 N) with chlorhexidine (without a bur) and 430.85±178.22 N (with a range of 122.07-835.32 N) with chlorhexidine (with a bur) (Table 1).

**Table 1:** The fracture resistance values of primary anterior teeth in different groups

**Groups**	**Minimum**	**Maximum**	**Mean force**	**Standard deviation**
**NaOCl without bur**	240.17	670.32	376.0769	107.28730
**NaOCl with bur**	157.67	1020.93	475.0461	186.89647
**Chlorhexidine without bur**	112.58	664.97	370.8810	175.45850
**Chlorhexidine with bur**	122.07	835.32	430.8471	178.21949

Two-way ANOVA showed that the type of irrigation solution (sodium hypochlorite and chlorhexidine) had no significant effect on fracture resistance values (P=0.47), but the effects of bur + irrigation on the fracture resistance were significant (P=0.02). In addition, the cumulative effect of the type of irrigation solution and bur application on fracture resistance was not significant (P=0.57).

In the non-bur preparation technique and application of sodium hypochlorite as an irrigation solution, 12 samples (46.2%) had an unfavorable fracture pattern (below the CEJ) and 14 (53.8%) had a favorable fracture pattern (above the CEJ). Additionally, in the application of chlorhexidine, 5 samples (25.0%) had an unfavorable fracture pattern (below the CEJ) and 15 samples (75.0%) had a favorable fracture pattern (above the CEJ) (Table 2).

**Table 1:** The fracture pattern of the samples in different methods of bur or non-bur preparation and sodium hypochlorite and chlorhexidine irrigation solutions

**Bur**	**Canal irrigation agents**	**Undesirable fracture pattern (below CEJ)**	**Desirable fracture pattern (above CEJ)**	**Total**
**Yes**	**NaOCl**	12 (46.2%)	14 (53.8%)	26 (100.0%)
	**Chlorhexidine**	5 (25.0%)	15 (75.0%)	20 (100.0%)
	**Total**	17 (37.0%)	29 (63.0%)	46 (100.0%)
**No**	**NaOCl**	9 (45.0%)	11 (55.0%)	20 (100.0%)
	**Chlorhexidine**	10 (35.7%)	18 (64.3%)	28 (100.0%)
	**Total**	19 (39.6%)	29 (60.4%)	48 (100.0%)
**Total**	**NaOCl**	21 (45.7%)	25 (54.3%)	46 (100.0%)
	**Chlorhexidine**	15 (31.2%)	33 (68.8%)	48 (100.0%)
	**Total**	36 (38.3%)	58 61.7%)	94 (100.0%)

In the bur preparation technique and application of sodium hypochlorite as an irrigation solution, 9 samples (45.0%) had an unfavorable fracture pattern (below the CEJ) and 11 samples (55.0%) had a favorable fracture pattern (above the CEJ). In this method and with application of chlorhexidine, 10 samples (35.7%) had an unfavorable fracture pattern (below the CEJ) and 18 (64.3%) had a favorable fracture pattern (above the CEJ) (Table 2).

Table 2 shows the fracture patterns of samples in different methods of bur or non-bur preparation and irrigation of the canals with sodium hypochlorite and chlorhexidine solutions.

## Discussion

Fracture of tooth-colored restorations is the main cause of treatment failure in the restoration of severely damaged primary anterior teeth. Therefore, achieving a proper retention of restoration is very important. Among the tooth-colored materials, composite resin is a routine option in anterior teeth due to its strength, abrasion resistance and aesthetic outcomes.^10^

Each irrigation solution in the present study has several properties. Sodium hypochlorite solution has high antimicrobial activity and dissolves tissues; it is used commonly in root canal therapy due to its affordability. Chlorhexidine, as a root canal irrigation agent, has a higher antibacterial activity and is also less toxic than NaOCl.

According to the results of this study, the fracture resistance rate of primary anterior teeth was 370.88±175.46 N in the presence of chlorhexidine without a bur and 430.0±178.8522 N in the presence of chlorhexidine with a bur. Chlorhexidine is able to compensate for the decrease in resin‒dentin bond strength observed in most cases of conventional adhesives and after long-term storage in water, and to preserve the morphological characteristics of the hybrid layers by inhibiting host protease.^11^

Edermir et al^12^ reported that endodontic irrigation with chlorhexidine induced a significant increase in the bond strength to root dentin. The possible effects of chlorhexidine in this field are related to the release of positively charged molecules at the levels treated with CHX and its ability to adsorb to the oral cavity surfaces.^13^ Theoretically, this process also occurs in exposed demineralized collagen fibrils and is a fundamental reason for maintaining the bond strength values after long-term storage in water. However, CHX might be adhered to high-speed collagen fibrils and can develop an adequate guarantee for this adhesion. In a study by Kim and Shin (2012), the use of chlorhexidine had no obvious effect on the bond strength of the samples.^14^ According to the results of the research, the mean fracture resistance of the teeth was 376.08 N under sodium hypochlorite solution irrigation without a bur and 475.05 N under sodium hypochlorite solution irrigation with the use of a bur.

Sodium hypochlorite solution, as an excellent non-specific proteolytic agent, is able to dissolve organic compounds and increase the penetration of composite monomers in the demineralized dentin structure.^15-17^ Sodium hypochlorite solution along with resin materials caused some problems and gave rise to a dentin surface covered with a rich oxygen layer, which interfered with the penetration of resin into tubular and intertubular dentin.^18-20^ This antibacterial agent could jeopardize the polymerization of bonding resins due to the oxidative effects of NaOCl and its derivatives.^13,21^ These products can prevent adhesive polymerization and cause dentin surface contamination. Another hypothesis in this regard is the removal of collagen fibers from the dentin surfaces following the use of NaOCl, which prevents the formation of a sound hybrid layer.‏

Comparison of similar experimental results regarding the fracture resistance values of teeth yields contradictory results because these results can be affected by many variables such as tooth condition before extraction, dental age, dental care conditions, pulpal position during tooth extraction, root anatomy and its dimensions, force angle, and tooth position.^22^

In summary, sodium hypochlorite solution leads to the oxidation of some components in the dentin matrix and formation of protein radicals. These materials compete with free radicals generated during photoactivation of resins, resulting in the cessation‏ of the polymeric chain‏ formation and incomplete polymerization processes.^19,23^ Microscopic studies also showed that sodium hypochlorite solution results in the removal of mineral-free collagen. The reduction of the bond strength of sodium hypochlorite cannot be attributed to incomplete dentin deproteinization.^24^ The presence of the smear layer impairs the adhesion mechanism and causes a decrease in bond strength; it has been shown that 2% chlorhexidine and 1% sodium hypochlorite are unable to completely remove the smear layer and there is no difference between the functions of the two agents in the smear layer removal.^24^

On the other hand, sodium hypochlorite solution removes dentinal organic components, including collagen, thereby increasing the penetration of monomers into the demineralized dentin structure. After application to dentin surfaces, sodium hypochlorite is broken down into oxygen and sodium chloride, and the oxygen obtained from these chemical components prevents complete polymerization of the resin bonding materials.^18,25^ The formation of oxygen bubbles in the interspace areas also interferes with the penetration of resin into tubules and intertubular dentin.

In a study by Bitter et al,^26^ the use of various irrigation methods, including sodium hypochlorite and chlorhexidine, had no distinct effect on the bond strength of different self-etch adhesive systems.^26^ On the other hand, some researchers have shown that the use of chlorhexidine had no negative effect on the immediate and prolonged bond strengths during cementation procedure of the post space.^27-29^

According to the results of this study, use of chlorhexidine in comparison with sodium hypochlorite solution had no significant effect on the changes in fracture resistance of the anterior teeth after post space preparation.

On the other hand, the use of bur + irrigation, compared with irrigation alone, had a significant effect on increasing the fracture resistance values of primary anterior teeth, which could be related to surface roughness following the application of a bur and an improvement in retention.

According to the present results, the favorable fracture frequency was higher with the use of chlorhexidine than NaOCl, so that 53.8% of the samples had a favorable fracture pattern (above the CEJ) in the non-bur and sodium hypochlorite solution method and 75.0% had a favorable fracture pattern in the application of chlorhexidine; in addition, 55% had a favorable fracture pattern (above the CEJ) with the use of a bur with sodium hypochlorite solution, and 64.3% had a favorable fracture pattern (above the CEJ) with the use of chlorhexidine.

There are limitations for in vitro studies due to the examination of human teeth with distinct dimensions and the application of compressive forces at a specific angle. In the oral environment, restored teeth are exposed to various variables such as continuous exposure to moisture, thermal fluctuations and pH, resulting from various foods, and exposure to bacteria and enzymes and are simultaneously affected by bite forces.‏ All these variables have obvious and undeniable effects on the tooth root and post retention and bond strength and can affect the clinical function of the posts. Accordingly, evaluation of the effect of variables on the tooth root‒post bond strength can be considered in future research.‏ Additionally, the assessment of long-term clinical durability and efficacy of the posts can reveal more details of their potential advantages and disadvantages.

## Conclusion

The results of this study showed that the type of irrigation solution had no significant effect on the fracture resistance but the effect of bur + irrigation was significant on the fracture resistance of the bonds, so that the fracture resistance of the samples significantly increased with the use of a bur + irrigation solution.

## Conflict of Interests

The authors claim to have no conflict of interest and take responsibility for the content of the manuscript.

## Authors’ Contributions

BS: design of the study, interpretation of data, revising the paper. SG: design of the study, performing the analysis, collection and interpretation of data, revising the paper. EN: Analysis and interpretation of data, Revising paper. FA: Collection of data, Contribution in data analysis, Writing paper. RK: Design of the work, interpretation of data, writing and revising the paper.

## Acknowledgments

Hereby the funding and support of the Research Deputy of Tehran University of Medical Sciences are highly appreciated.

## Funding

Research Deputy of Tehran University of Medical Sciences

## Ethics Approval

There was no particular problem in terms of observing ethical considerations regarding laboratory conditions of the research and the absence of any contact to the patients.
